# The discovery of a novel knockdown resistance (*kdr*) mutation A1007G on *Aedesaegypti* (Diptera: Culicidae) from Malaysia

**DOI:** 10.1038/s41598-021-84669-w

**Published:** 2021-03-04

**Authors:** Wan Fatma Zuharah, Maryam Sufian

**Affiliations:** 1 School of Biological Sciences, Universiti Sains Malaysia, 11800 Minden, Penang Malaysia; 2 Vector Control Research Unit, School of Biological Sciences, Universiti Sains Malaysia, 11800 Minden, Penang Malaysia

**Keywords:** Molecular biology, Diseases

## Abstract

The usage of insecticide rendered the successful vector control program with the high usage of the pyrethroid. However, the intensive and extensive use of pyrethroid, causing resistance in *Aedes* *aegypti* and hampered the control program. Knockdown resistance (*kdr*) resulting from the Voltage-Gated Sodium Channel (VGSC) is one of the mechanisms of resistance in pyrethroid group insecticide. Investigating the phenotypic status of *Ae.* *aegypti* mosquitoes is a lead in knowing the current resistance status and as an indicator of the genotypic resistance. In this study, we investigate the resistance in phenotypic and genotypic of *Ae.* *aegypti* with a new *kdr* mutation point A1007G was detected. Using the adult bioassay, we tested the phenotypic resistance from the Selangor state against 0.75% permethrin, 0.05% deltamethrin with and without the addition of PBO synergist. Permethrin-resistant and deltamethrin-resistant, including susceptible samples, were subjected to genotyping analysis on mutation point in domain II and domain III of Voltage-Gated Sodium Channel (VGSC). Adult bioassay revealed that the *Ae.* *aegypti* was highly resistance toward 0.75% permethrin and 0.05% deltamethrin. The bioassay with the presence of PBO synergist showed an increment of mortality rate, but *Ae.* *aegypti* status is still resistance towards both insecticides. Genotyping result showed that three common *kdr* mutations (S989P, V1016G, and F1534C) have existed in the *Ae.* *aegypti* population. A new novel mutation on A1007G was also detected in this population, which is the first time reported. This study has brought a piece of information on the current resistance status in *Ae.* *aegypti* in Malaysia. The detection of new mutation point of A1007G has added the knowledge on the resistance in mosquitoes. Thus, this study will aid with the decision making in the usage of insecticides in the vector control program; before this invaluable insecticide rendered ineffective in killing mosquitoes.

## Introduction

Dengue is the most important arboviral infections of humans transmitted by *Aedes* mosquitoes^1^. *Aedes* *aegypti* (Linnaeus) is extensively known as the primary vectors of dengue viruses (DENVs), with *Aedes* *albopictus* (Skuse) regarded as a secondary vector^2^. DENV in humans is often life-threatening and can lead to a range of clinical manifestations from mild fever to potential dengue shock syndrome in its most severity^3^. In Malaysia, by November 2020, 83,972 cases have been reported with 120 deaths with the highest recorded in Selangor state^4^.

The use of chemical insecticides is still a key component of *Aedes* vector control method by targeting the adult mosquitoes, either through an indoor residual spray, treated bed nets or space spraying once larval source reduction fails to reduce the population of adult mosquitoes or adults become a serious nuisance^5,6^. The heavy reliance on using residual insecticides such as through intensive and prolonged use have caused insecticide resistance in *Aedes* mosquitoes, and reduce the effectiveness of adulticide-based control programs around the world, including Malaysia^7–9^.

Organochlorines, organophosphate, carbamates and pyrethroids are four major categories of insecticides used in vector control programmes. Though, pyrethroid insecticide is extensively used on insect pests in vector control or agriculture sectors worldwide due to its fast-acting and relatively low mammalian toxicity^10,11^. In Malaysia, insecticides from the pyrethroid class such as permethrin and deltamethrin are mainly used for adulticiding purpose in the vector control programme by the Ministry of Health. Thus, the intense usage of these insecticides has been reported to cause insecticide resistance of *Ae.* *aegypti* populations in Malaysia.

Insecticide resistance can derive from different mechanisms, the main ones being modifications in the target sites, known as target site resistance. In general, pyrethroids function as neurotoxins that target voltage-gated sodium channels (VGSC) and interfere electronic signalling in the nervous system, which results in paralysis and death of the mosquito, an effect often referred to as “knockdown”^12,13^. Target site resistance in mosquitoes is related to either single or multiple mutations in target genes; for example, the VGSC gene leads to knockdown resistance (*kdr*), mutations in the acetylcholinesterase (Ace-1) gene and GABA receptors^14^. However, *kdr* is the most well-studied target-site pyrethroid resistance mechanisms in insects, including disease vectors such as against *Ae.* *aegypti* mosquitoes^15^ and a good predictor of the efficacy of pyrethroids is through genotyping of *kdr* mutant alleles^16^.

Mutations at three codon positions of the VGSC gene (S989P, I1011M/V and V1016G/I) have been primarily reported in domain II^17^ while mutation at the position of F1534C was reported in domain III^18^ of the pyrethroid-resistant *Aedes* populations worldwide. In South-East Asia, the mutation in the position of F1534C and V1016G of the VSGC was reported in the pyrethroid-resistant *Ae.* *aegypti* in Malaysia^17^. Some of these mutations, such as the F1534C, have also been reported in neighbouring countries such as in Singapore^15,18^ and Thailand^19^. Whereas, mutations at I1011V and V1016I were identified in Latin American countries^20^. Whereas, four novel mutant alleles at F1269C, T1520I, F1534S, and F1534L on domain III has been found in Vietnam^21^, India^22^, and China^23^ respectively. Besides, a new pyrethroid mutation (V410L) in domain I have been recently detected in Brazil^24^.

Thus, it is very likely for the emergence of new *kdr* mutations when pyrethroids remained the primary insecticide-based interventions in the control of *Ae.* *aegypti.* These detections act as a crucial role in resistance management of *Ae.* *aegypti* by observing the occurrence of pyrethroid resistance. By understanding the mechanisms of resistance, appropriate insecticides can be used for effective control of target vector species. In this study, we investigated the susceptibility of *Ae.* *aegypti* on the pyrethroid resistance and examined the *kdr* mutations in this mosquito species at dengue-endemic areas in Selangor, Malaysia. To the best of our knowledge, this study is the first to report the presence of a new novel mutation A1007G in *Ae.* *aegypti* Selangor strain.

## Materials and methods

### Mosquito strains

The sampling site was conducted in Selangor, which is at the centre of Peninsular Malaysia. Mosquito samples were collected from the urban area located at Puncak Perdana (3° 07′ 57.3 ″N 101° 29′ 35.0″ E) UiTM campus consisted of a university student hostel. This hostel at Puncak Perdana, Selangor is surrounded by greenery with plenty of undeveloped lands and on-going developments. These sampling sites were chosen because the number of dengue cases is highly reported by a previous study^25^.

### Collection and maintenance of *Aedes* mosquitoes

Sample collection was done by using ovitraps to collect eggs and larvae of mosquitoes. The traps were placed close to students’ residential premise, and near vegetation to collect both *Aedes* species. Approximately 50 ovitraps were randomly set up for each sampling locations and were collected after 5 days introduced to the field. All water containing larvae and wooden paddles with eggs were brought back to Medical Entomology Laboratory, Universiti Sains Malaysia to be rear as parent generation, F_0_. Mosquito populations were maintained at a temperature of 25 ± 2 °C and relative humidity (RH) of 52 ± 2%.

### Permethrin and deltamethrin adult bioassay on insecticide susceptibility test

Adult bioassays were carried out according to the standard WHO protocol^26^. All strains were tested against pyrethroid insecticides in four replicates of 25 non-blood fed female mosquito ranging from 3 to 5 days old. The insecticides used were; (1) 0.75% permethrin (type I), (2) 0.05% deltamethrin (type II), (3) PBO + 0.75% permethrin, and (4) PBO + 0.05% permethrin. Due to high resistance usually occurred in *Aedes* mosquito in Malaysia, *Anopheles* diagnostic dose was used in this study. Furthermore, the *Aedes* diagnostic dose is still tentative dose currently^27^. Controls were done in two replicates for each insecticide using silicone oil for pyrethroid control. While the Vector Control Research Unit (VCRU), Universiti Sains Malaysia susceptible strain was used as a reference for baseline susceptible study towards permethrin and deltamethrin insecticides.

Mosquitoes in the holding tubes were given 1-h acclimation time. Any damaged, injured, and dead mosquitoes were replaced with the healthy ones after the acclimation time. The mosquitoes from the holding tube were then transferred into the test tube containing treated impregnated paper. Knockdown data were then recorded every 5 min for an interval of 1 h once exposed to insecticides. After the exposure period, mosquitoes were immediately transferred back into the holding tubes with 10% sucrose solution soaked cotton wools as a food source. The mortality rate was determined after 24 h. Percentage of mortality after 24 h exposure to insecticides in the adult bioassay test was characterized for the susceptibility status using WHO criteria where mosquitoes are considered as: (1) susceptible, if the percentage mortality is between 98 and 100%, (2) incipient resistant if mortality is between 90 and 97% and (3) resistant if mortality is < 90%^26^.

Mosquito strains that were highly resistant to all insecticide treatments were also tested using insecticide synergist, PBO to assess the involvement of cytochrome P450s in the insecticide resistance mechanisms. In this case, both permethrin and deltamethrin were found highly resistance. Mosquitoes were pre-exposed to 4% PBO for 1 h before directly exposed to the mentioned insecticides. In parallel, controls were done using bioassays with PBO 4% and exposing the mosquitoes to control papers before transferring to insecticide tubes. All treatments were replicated four times with two sets of control for each insecticide, with and without synergist associated. Overall, alive and dead *Ae.* *aegypti* samples from Selangor strain (of each insecticide treatment with and without synergist) from the 24 h recovery period was kept in 1.5 ml microcentrifuge tube and immediately stored in -20 °C until DNA extraction.

### Genomic DNA extraction

Homogenate and mosquito DNA isolation from individual mosquitoes were prepared following GENEzol Reagent of Geneaid protocol, with slight modification during the extraction method on the amount of reagent used. A total of 100 µl of GENEzol reagent was inserted into 1.5 ml of microcentrifuge tube and homogenized using a sterile plastic grinder until disintegrated. The sample was then incubated for 5 min at room temperature, and transfer to a new microcentrifuge tube. Following the phase separation step, 20 µl of chloroform was added into the sample, shake vigorously for 10 s and centrifuged at 14,000×*g* for 15 min at 4 °C to separate the phases. The upper aqueous phase was then transferred to a new 1.5 ml microcentrifuge tube. Briefly, during the DNA precipitation, 30 µl of absolute ethanol was added into the sample, mixed by inverting the tube several times, incubated for 5 min at room temperature then centrifuged at 2000×*g* for 5 min at 4 °C. Subsequently, the supernatant was then carefully removed. Afterwards, 100 µl of 0.1 M sodium citrate in 10% ethanol was added into the sample and incubated for 30 min at room temperature. The tube was occasionally inverted during incubation. The sample was then centrifuged at 2000×*g* for 5 min at 4 °C, and the supernatant was removed. The above wash step was repeated once more. Then, 150 µl of 70% ethanol was added into the sample and was incubated for 15 min at room temperature, while the tube was gently inverted occasionally. The sample was centrifuged at 2000×*g* for 5 min at 4 °C, and the supernatant was carefully removed. The DNA pellet was then air-dried for 10 min at room temperature. During DNA resuspension step, 30 µl of TE Buffer was added to the DNA pellet. The DNA sample was then incubated at 60 °C for 15 min and centrifuged at 14,000×*g* for 15 min at 4 °C. 30 µl of the supernatant containing DNA was then transferred to a new 1.5 ml microcentrifuge tube and stored at − 20 °C until PCR analysis.

### Detection of knockdown resistance mutation (*kdr*) in domain II and domain III of VGSC gene in *Aedes aegypti*

To identify potential *kdr* mutations, two fragments of the coding region of the VGSC gene spanning exon 19 to exon 31 (covering the 989, 1011, 1016, 1007 and 1534 coding positions) were amplified from DNA samples and directly sequenced. PCR mix of total 25 μl was prepared using 12.5 μl of EconoTaq Plus Green 2X Master mix, 0.25 μl of Forward primer, 0.25 μl of Reverse primer, 1 μl of DNA template and lastly 11 μl of dH2O on ice. To ensure even mixture of the PCR mix, the PCR mix was centrifuged for 10 s using a mini centrifuge machine before subjected to PCR analysis.

A total of two sets of PCR mix were prepared for each DNA sample, (1) for domain II (mutations at S989P, I1011M/V, L1014F, V1016G/I, and A1007G) the initial fragment amplification was performed by using primers AaSCF1 (AGACAATGTGGATCGCTTCC) and AaSCR4 (GGACGCAATCTGGCTTGTTA)^28^, while (2) for domain III primers AaSCF7 (GAGAACTCGCCGATGAACTT) and AaSCR7 (GACGACGAAATCGAACAGGT) were used in the polymerase chain reaction to detect F1534C mutation^28^. The PCR parameters were set at 94 °C for 5 min for initial denaturation, 35 cycles of 94 °C for 30 s for denaturation, 57 °C for 30 s for annealing and 72 °C for 1 min extension, followed by final elongation step at 72 °C for 10 min and was set to hold at 4 °C for ∞ ^18,28^.

The PCR products were size separated on a 1.5% agarose gel stained with nucleic acid (Healthview). The gel containing size separated PCR products were visualized by using a gel documentation system by BioRad (Gel Doc XR + System). The PCR products were prepared and sent for purification and sequencing to MyTACG DNA Sequencing Services. The DNA sequencing was carried out by using primers AaSCF3 (GTGGAACTTCACCGACTTCA) and AaSCR6 (CGACTTGATCCAGTTTGGAGA) for domain II and AaSCR8 (TAGCTTTCAGCGGCTTCTTC) for domain III of *Ae.* *aegypti*^28^.

### Statistical analysis

The statistical analysis was done using probit analysis from IBM SPSS Statistic Version 24 to find the knockdown time of 50% and 95% of the tested population (KdT50 and KdT95). Based on the KdT50 and KdT95 values, the resistance ratio (RR) was calculated as in the formula below:

$$RR=\frac{KdT50/95\, of\, the \,field\, strain}{{KdT50/95}\,of\, the\, susceptible \, strain}$$

Based on the RR value, the field population is considered as susceptible when the RR < 5, mosquitoes are considered to have a moderate resistance when RR is between 5 and 10, and the mosquitoes are highly resistant when RR > 10 ^26^.

In contrast, the percentage of mortality of the tested population was recorded after 24 h by using the formula as follows:

$$Observed\, mortality=\frac{Total\, number\, of\, dead\, mosquitoes}{Total\, sample\, size}\times 100$$

Abbott`s formula is used as the corrective formula if the mortality rate of control replicates ranged between 10 and 20%. However, the test was discarded and repeated if the percentage in control mortality was more than ≥ 20%. Abbott’s formula was not applied in this study.

$$Corrected\, mortality= \frac{\% Observed\, mortality-\% Control\, mortality}{100-\% Control\, mortality} \times 100$$

The sequences obtained from the sequencing company (My TAGC DNA Sequencing Services) were aligned using ClustalW and translated into a protein sequence by using protein translation tool from Mega *v*7.0 software^29^.

### Ethic declarations

The authors declare no competing interests. Each author has approved the submitted version (and any substantially modified version that involves the author's contribution to the study) and have agreed both to be personally accountable for the author's own contributions. The funders did not have any additional role in the study design, data collection and analysis, decision to publish, or preparation of the manuscript.

## Results

### Susceptibility status and resistance pattern of *Aedes aegypti* in Selangor, Malaysia

The results of the adult insecticide bioassays conducted on the WHO lethal dose of 0.75% permethrin for *Ae.* *aegypti* population after the 1-h test indicated that Selangor strain exhibited the highest ${\mathrm{KdT}}_{50}$ (932.97 min) and ${\mathrm{KdT}}_{95}$ (87,871.68 min) when exposed to permethrin with the RR_50_ at 58.93 fold and RR_95_ at 4045.66 fold respectively compared to 0.05% deltamethrin. The exposure of *Ae.* *aegypti* towards 0.05% deltamethrin showed significantly lower ${\mathrm{KdT}}_{50}$ (252.21 min) and ${\mathrm{KdT}}_{95}$ (2305.89 min) with RR_50_ value of 13.64 fold and RR_95_ 90.00 fold, respectively. Thus suggested that *Ae.* *aegypti* Selangor strain has developed significantly higher resistance towards 0.75% permethrin in comparison to 0.05% deltamethrin (Table 1).

**Table 1:** Knockdown time (min) ${\mathrm{KdT}}_{50}$ and ${\mathrm{KdT}}_{95}$ of 1-h exposure time towards two different types of WHO insecticide treatments (permethrin 0.75% and deltamethrin 0.05%) and PBO synergist against *Aedes* *aegypti* female adults for susceptible VCRU lab strain and Selangor field strain.

Strain	Insecticide	${\mathrm{KdT}}_{50}$ (min) (lower bound–upper bound)	RR_50_	${\mathrm{KdT}}_{95}$ (min) (lower bound–upper bound)	RR_95_	Regression equation
VCRU	Permethrin	15.83 (15.22–16.42)	1.00	21.72 (20.60–23.29)	1.00	y = − 10.76x − 14.38
	Deltamethrin	18.49 (17.84–19.12)	1.00	25.62 (24.39–27.30)	1.00	y = − 12.32x − 14.71
Selangor	Permethrin	932.97 (325.77–13,123.18)	58.93	87,871.68 (7820.20–411,111,614.28)	4045.66	y = − 8.76x − 2.48
	Deltamethrin	252.21 (151.65–715.08)	13.64	2305.89 (786.70–21,552.03)	90.00	y = − 8.38x − 4.11
	Permethrin + PBO	129.11 (98.47–200.95)	8.15	888.93 (463.80–2643.48)	40.93	y = − 10.24x − 4.14
	Deltamethrin + PBO	52.29 (48.52–57.64)	2.82	113.87 (94.02–153.06)	4.44	y = − 14.09x − 8.36

${KdT}_{50}$ knock down times required to kill 50% of the population exposed, ${KdT}_{95}$ knock down times required to kill 95% of the population exposed.

Interestingly, the exposure of *Ae.* *aegypti* with insecticide and PBO is significantly lower compared to treatment without PBO (Table 1). The 0.75% permethrin + PBO treatment indicates significantly lower ${\mathrm{KdT}}_{50}$ (129.11 min) and ${\mathrm{KdT}}_{95}$ (888.93) with RR values of 8.15 fold and 40.93 fold respectively as compared to 0.75% permethrin treatment only. While the RR of *Ae.* *aegypti* with 0.05% deltamethrin + PBO was with RR_50_ value of 2.82 fold and RR_95_ 4.44 fold. This suggested that PBO might play a role in the resistance status of *Ae.* *aegypti* population in Selangor.

The 24 h mortality results had confirmed that the susceptible VCRU lab strain as reference strain is still fully susceptible to both 0.75% permethrin and 0.05% deltamethrin insecticides with 100% mortality (Table 2). On the other hand, Selangor strain is strongly resistant towards both type I and type II pyrethroids as defined by WHO; < 90% mortality indicates resistance (WHO 2016). Surprisingly, a high resistance level could be observed for *Ae.* *aegypti* towards deltamethrin 0.05% with only 13% mortality after 24 h exposure period. In contrast, 27% mortality was observed on *Ae.* *aegypti,* when exposed to permethrin 0.75% 24 h post-exposure mortality, indicates resistance (Table 2).

**Table 2:** Resistance status of *Aedes* *aegypti* female adults after 24 h exposure period towards two different types of WHO insecticide treatments (permethrin 0.75% and deltamethrin 0.05%) and PBO synergist for susceptible VCRU lab strain and Selangor field strain.

Strain	Insecticide	% Mortality ± SE (n)	Status
VCRU	Permethrin	100 ± 0 (100)	S
	Deltamethrin	100 ± 0 (100)	S
Selangor	Permethrin	27 ± 1.44 (100)	R
	Deltamethrin	13 ± 0.85 (100)	R
	Permethrin + PBO	41 ± 2.87 (100)	R
	Deltamethrin + PBO	49 ± 1.31 (100)	R

S indicated susceptible and R indicated resistance.

For the synergist assay, pre-exposure to PBO induced a partial recovery of susceptibility for both deltamethrin and permethrin insecticides with 49% and 41% mortality in *Ae.* *aegypti* but still within the resistance range (Table 2). Overall, an increase in mortality was observed towards both insecticides with synergist compared between assays without synergist suggested that cytochrome P450 monooxygenases play a predominant role in the resistance of *Ae.* *aegypti* population.

### Searching for potential *kdr* mutation in *Ae. aegypti* associated with pyrethroid resistance

**Figure 1:**
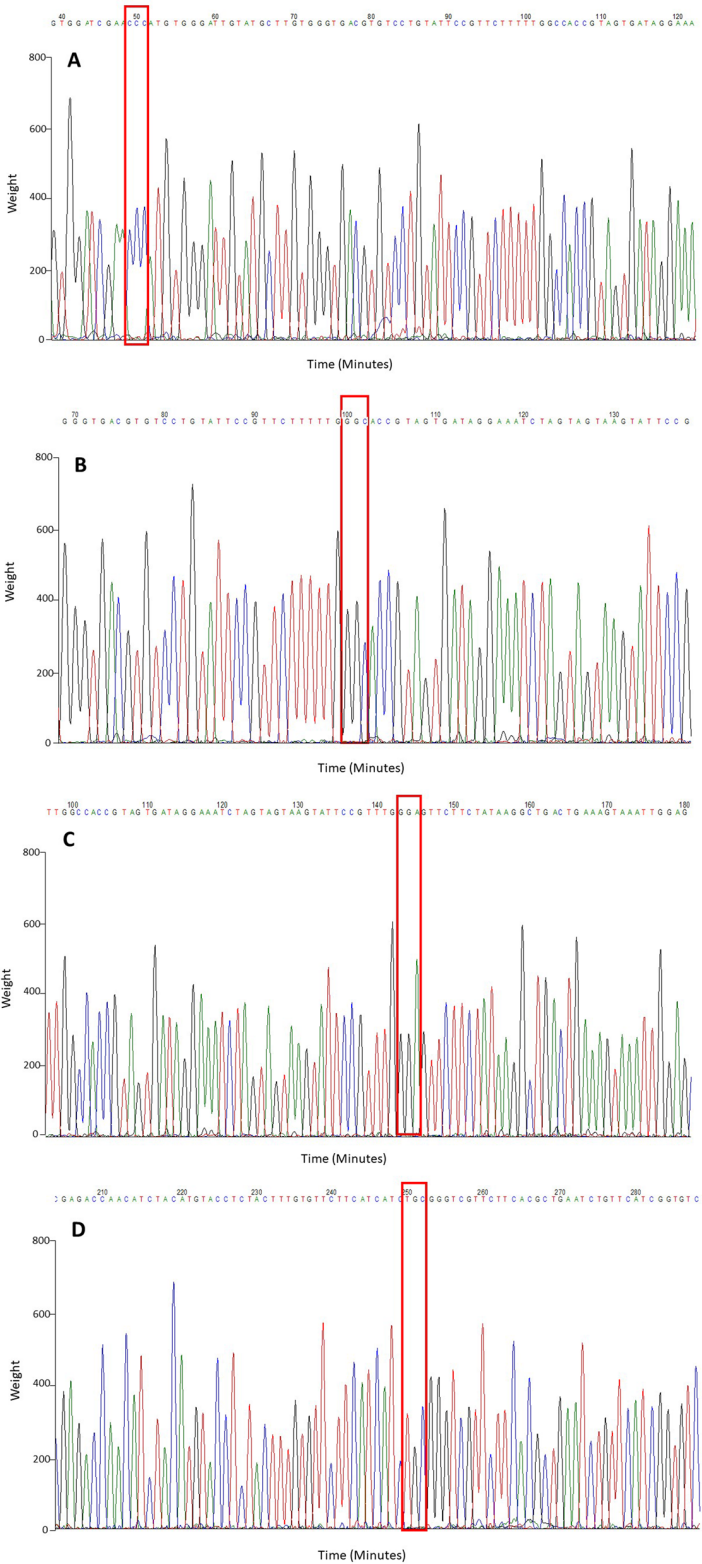
Chromatograph of sequences at a position of homozygous *kdr* resistance in VGSC domain II regions of (**A**) S989P, (**B**) A1007G (a new novel mutation), (**C**) V1016G, and domain III region of (**D**) F1534C which related to pyrethroid-resistance. A red box indicated the position of the amino acids.

**Figure 2:**
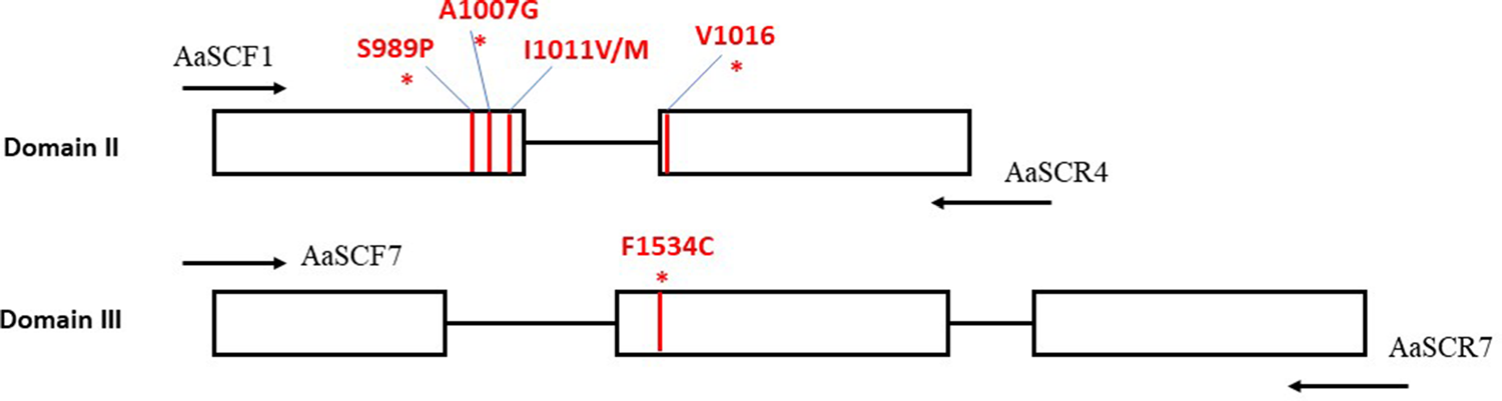
The *kdr* mutations (red lines) and primers (arrows) are indicated in the partial genomic region encoding Voltage-gated Sodium Channel (VGSC). Primer AaSCF1 and AaSCR4 were used to amplify VGSC domain II, meanwhile primers AaSCF7 and AaSCR7 for domain III. The four *kdr* mutations of S989P, A1007G (a new novel mutation), V1016G dan F1534C were found in the Selangor, Malaysia strain marked by red asterisks.

DNA sequenced of the partial domain II and domain III of the VGSC gene of *Ae.* *aegypti* Selangor population revealed the presence of four mutations at segment 6 of domain II and domain III. Three mutations were present in domain II particularly at the position of S989P and V1016G which were due to the substitution on the first codon (TCC to CCC) and second codon at (GTA to GGA), respectively. A new mutation was also detected at the position of A1007G due to substitute of the second codon (GCC to GGC). The mutation A1007G is the first reported novel mutation of domain II found in *Ae.* *aegypti* mosquito population in this study that resistance toward pyrethroid represents by Alanine (GCC) to Glycine (GGC) (Fig. 1). Other than domain II, mutation F1534C present at domain III was due to T > G substitution on the second substitution of the codon, leading to Phenylalanine (TTC) to Cysteine (TGC) mutation also found in this study. The location of *kdr* mutations and primers are illustrated in Fig. 2.

In the course of study, a total of 26 selected *Ae.* *aegypti* samples that confirmed resistance in bioassay study were sequenced for partial domain II and domain III. A total of none individuals were homozygous susceptible S989 (encoded as S/S), 17 were homozygous resistant P989 (encoded as P/P), nine were homozygous susceptible V1016 (encoded as V/V), 17 were homozygous resistant G1016 (encoded as G/G), four were heterozygous A1007G (encoded as A/G), one was homozygous resistant G1007 (encoded as G/G), 17 were homozygous susceptible F1534 (encoded as F/F), and nine were homozygous resistant C1534 (encoded as C/C) which were found present in the sequences (Table 3).

**Table 3:** Frequencies of S989P, V1016G, A1007G and F1534C substitution in parental of the VSGC gene in *Aedes* *aegypti* Selangor strain.

Insecticide	N	Phenotype	Genotype												Resistance allelic frequency			
			S989P			V1016G			A1007G			F1534C						
			SS	SP	PP	VV	VG	GG	AA	AG	GG	FF	FC	CC	S/P	V/G	A/G	F/C
Permethrin	5	R	1 (0.2)	0	4 (0.8)	2 (0.4)	0	3 (0.6)	5 (1)	0	0	4(0.8)	0	1(0.2)	0.8	0.6	0	0.2
	3	S	–	–	–	–	–	–	–	–	–	–	–	–	–	–	–	–
Deltamethrin	5	R	0	0	5 (1)	0	0	5 (1)	5 (1)	0	0	5(1)	0	0	1	1	0	0
	3	S	–	–	–	–	–	–	–	–	–	–	–	–	–	–	–	–
Permethrin + PBO	5	R	3 (0.6)	0	2 (0.4)	2 (0.4)	0	3 (0.6)	3 (0.6)	2 (0.4)	0	2(0.4)	0	3(0.6)	0.4	0.6	0.2	0.6
	3	S	3 (1)	0	0	3 (1)	0	0	1 (0.3)	2 (0.67)	0	0	0	3(1)	0	0	0.34	1
Deltamethrin + PBO	5	R	1 (0.2)	0	4 (0.8)	1 (0.2)	0	4 (0.8)	4 (0.8)	0	1 (0.2)	4(0.8)	0	1(0.2)	0.8	0.8	0.3	0.2
	3	S	1 (0.3)	0	2 (0.67)	1 (0.33)	0	2 (0.67)	3 (1)	0	0	2(0.67)	0	1(0.33)	0.67	0.67	0	0.33

**Figure 3:**
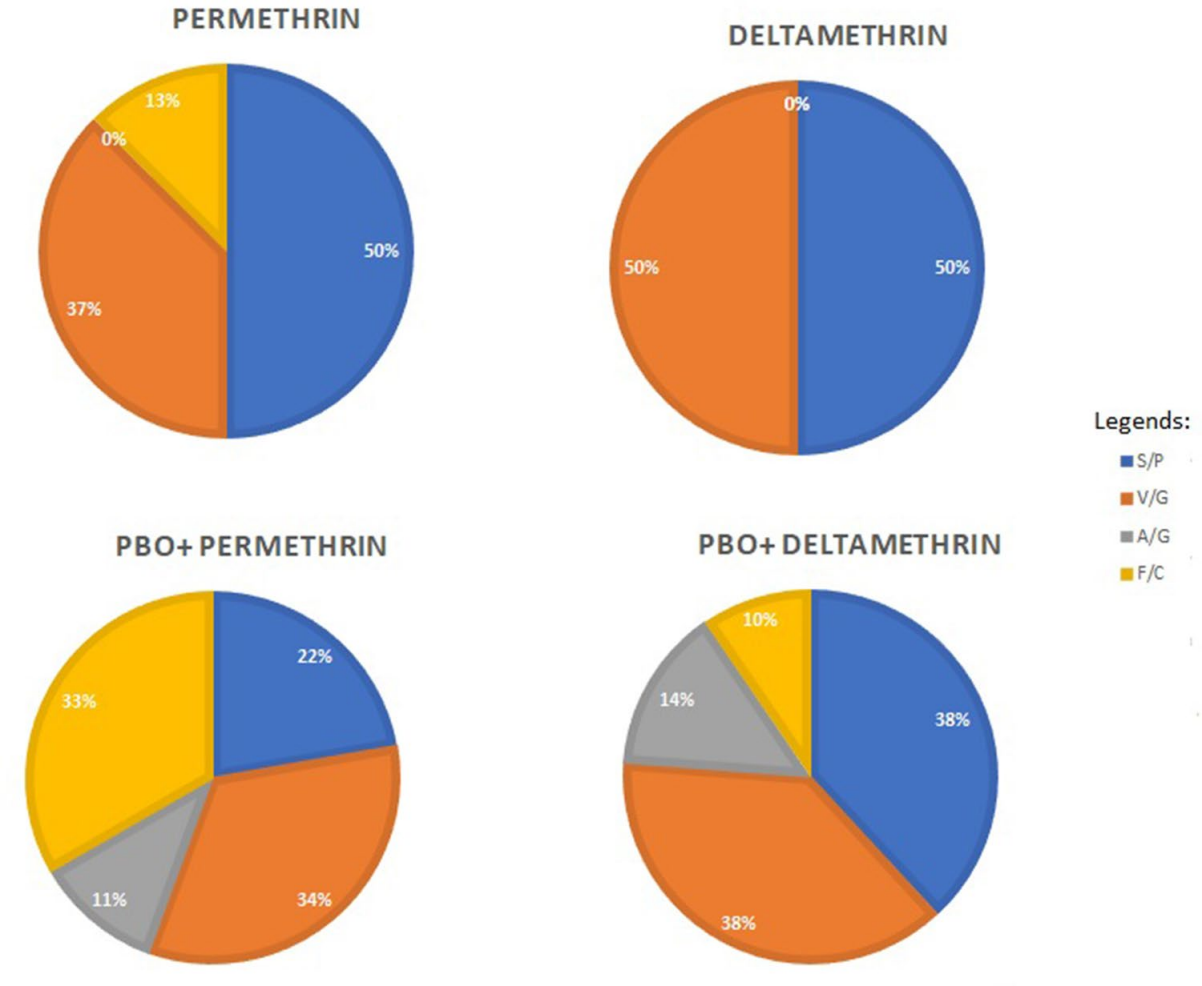
The resistance allelic frequency of *Aedes* *aegypti* Selangor strain identified with amino acid substitution in domain II and domain III of VGSC.

Overall, we detected three patterns of co-occurrence of point mutations; S989P/V1016G, A1007G/F1534C, and V1016G/A1007G/F1534C in *Ae.* *aegypti* strain. Based on Table 3, the frequency of the mutation S989P for permethrin-resistant individuals was the highest with 0.8. Besides, the permethrin-resistant individual has the lowest frequency of 0.2 mutant alleles 1534C. Meanwhile, deltamethrin-resistant individuals had similar frequencies of one for the mutant allele 989P and 1016G. No *kdr* resistance alleles in positions 1007 and 1534 were detected in deltamethrin-resistant individuals (Fig. 3). Besides, permethrin-susceptible and deltamethrin-susceptible individuals were not selected for analysis due to the low sample size and poor DNA quality of the extracted individuals. Hence, only the best PCR product and the best sequence quality were used for the analysis.

Permethrin-resistant strains with PBO synergist showed the highest frequencies of 0.6 for 1016G and 1534C mutant alleles, while with the lowest frequency of 0.2 mutant allele 1534C for deltamethrin-resistant with PBO synergist. For the susceptible strains with PBO synergist, zero frequencies of mutant 989P and 1016G were detected for permethrin. Meanwhile, similar frequencies of 0.67 mutant alleles 989P and 1016G were found for the deltamethrin with PBO synergist individuals (Table 3).

## Discussion

The development of pyrethroid resistance in the *Ae.* *aegypti* population has a potential in the failure of mosquito vector management. Although the insecticide is a primary component for the mosquito management, the unplanned and excessive use of it had led to the development of insecticide resistance^30,31^. A recent study has mapped the existence of resistance in *Ae.* *aegypti* on the main hotspot area in Selangor, Malaysia, highlighted the role of target site mutation profile in response to pyrethroid class (permethrin and deltamethrin).

*Aedes* *aegypti* in this study was found highly resistance towards both permethrin and deltamethrin. Eventhough, in the presence of synergist, which is Piperonyl butoxide (PBO) does not help reduce resistance status in *Ae.* *aegypti* in this study. Synergist PBO is used to suppresses the activity of detoxifying enzymes such as P450s and non-specific esterases that were found in pyrethroid resistance. Even after the suppression of the detoxifying enzyme, our results still exhibited resistance on pyrethroid. Five years back, the study showed the closest area to the study sites, Kuala Lumpur was moderate resistance towards permethrin and deltamethrin (87% and 89% mortality)^17^. Since the *Ae.* *aegypti* in Selangor was collected in the urban setting area, the selection pressure to insecticides is higher due to the high case of dengue via fogging^32^ or using household insecticides such as aerosol spray^33^. A pyrethroid is known as a “knockdown” insecticide that paralyzed the insect which responsible for the rection of sensitivity in the insect’s nervous system, and the confirmed mortality can be observed after 24 h. With the mortality of 27% (permethrin) and 13% (deltamethrin), the alive individual has developed resistance.

Generally, the four main insecticides have been used in vector management: organophosphate (OP), Organochlorine (OC), Carbamates (C) and pyrethroid (PY). Currently, OP and PY were applied for the residual spraying and space spraying, and only a limited number of insecticides can be targeted the adult stage of mosquitoes^34^. No new other insecticide has been approved since the last 15 years^34^, and more resistance has been recorded recently either on the *kdr* mutation or resistance enzyme.

Genotyping the *kdr* mutation in domain II (S989, I1011, L1014 and V1016) and domain III (F1534) were highly reported in *Ae.* *aegypti* worldwide^16,19^. Other than these common resistance mutations, V1023G has previously reported in Indonesia and Malaysia and has played a role in reducing sensitivity in VGSC^31^. Finding from this recent study, it clearly demonstrated a new detected mutation at the position A1007G in *Ae.* *aegypti*. The increase of the resistance frequencies, including this new mutation point in *Ae.* *aegypti* population might reduce the vector control program efficiency, which mostly relies on the pyrethroid insecticides.

In this study, we also detected the presence up to two to three mutations in one individual. The 1016G mutation is usually found with a 989P mutation as found by Srisawat et al.^35^. There are no proven to associate the S989P alone with the relation to pyrethroid resistance but coupling with other mutation sites can cause slightly synergistic effects and increase the resistance effects^36^. The study by Ishak^17^ stated that the increased resistance revealed the role of the V1016G mutation in Malaysia in the *Ae.* *aegypti* that possess the 1534C allele. However, the presence of A1007G coupling with F1534C resistance, or triple mutations (V1016G/A1007G/F1534C) were found in both deltamethrin and permethrin resistance samples. This mutation believed to reduce the VGSC sensitivity to both type I and type II pyrethroid due to the resistance phenotype represented in the study. The mutational effect of A1007G is only apparent if coupled with other mutation points. Thus, we are unable to conclude that this A1007G alone has caused the actual resistance to *Ae.* *aegypti*. However, this A1007G mutation can possibly increase the resistance effects on *Ae.* *aegypti* mosquitoes against pyrethroid. In addition, only A1007G was found heterozygous, but the rest of the mutations were detected as homozygous resistance. The homozygous resistance is associated with the fitness cost of haplotypic, which survived at a higher rate than heterozygous mosquitoes^37^.

According to Ahbi et al.^38^, high detection of F1534C mutation in *Ae.* *aegypti* was found closely related to pyrethroid resistance found in Malaysia, and her study concludes that domain III is the major point of mutations in Kelantan, Malaysia. The predominance of a resistance haplotype of F1534C in the spawning of *Ae.* *aegypti* supported the resistance of pyrethroid. This mutation point was also detected in *Ae.* *albopictus* in Singapore^18^. Thus, pyrethroid resistance might occur due to the presence of *kdr* mutation or the P450 monooxygenase metabolic resistance^39^.

The existence of a new *kdr* mutation gene in dengue vector may work as a warning sign of overused pyrethroid in the vector control program. It is crucial to screen the current susceptibility status of *Aedes* mosquitoes, especially the population in the dengue areas before this invaluable insecticide is rendered ineffective in killing the *Ae.* *aegypti*. Monitoring on mosquitoes' resistance status will aid in the decision of insecticide usage in the vector control program.
